# Acute Hemolytic Anemia and Immune Thrombocytopenia: A Rare Case of Parvovirus B19-Induced Evans Syndrome

**DOI:** 10.7759/cureus.88845

**Published:** 2025-07-27

**Authors:** Weiying Li, Sana Hussain, Azhar Hussain, George Everett

**Affiliations:** 1 Internal Medicine, AdventHealth Orlando, Orlando, USA; 2 Internal Medicine, Univeristy Hospital Galway, Galway, IRL

**Keywords:** antiphospholipid, antiphospholipid antibody syndrome (aps), autoimmune hemolytic anemia (aiha), evans syndrome, idiopathic thrombocytopenia purpura (itp), immune thrombocytopenia (itp), parvovirus-b19

## Abstract

Evans syndrome is a rare condition that can be seen among patients with pre-existing rheumatological disorders such as lupus, rheumatoid arthritis, or adult-onset Still's disease. There is an association between positive anti-phospholipid antibodies and the development of Evans syndrome, but the underlying pathophysiology remains unknown. To our knowledge, this is one of the few case reports to date that describes the development of Evans syndrome triggered by parvovirus B19 in patients with pre-existing positive antiphospholipid antibodies.

A 47-year-old female with a past medical history of positive antiphospholipid antibodies presented with a petechial rash on the bilateral lower extremities for two days. She reported that her child had slapped cheek disease recently. Physical examination revealed a rash on the bilateral lower extremities and mucosal petechiae in the oral cavity. She was otherwise hemodynamically stable. Initial labs showed normocytic anemia and critically low platelets of less than 2 x 103/uL. The reticulocyte count was low. Other labs showed elevated lactate dehydrogenase (LDH) and low haptoglobin, which was consistent with hemolysis. The direct antiglobulin test (DAT) was positive for warm autoantibody, which further confirmed autoimmune hemolysis. The peripheral smear showed normocytic anemia with severe thrombocytopenia with scattered spherocytes. Flow cytometry showed no evidence of lymphoma. Extensive work-ups for ADAMTS13, HIV, and hepatitis were negative. The IgM titer of parvovirus B19 was significantly elevated. The patient also had positive lupus anticoagulant and positive antibodies for beta 2 glycoprotein and cardiolipin, which were initially detected many years ago, but she never had thrombotic events or recurrent pregnancy loss that would fulfill the diagnosis of antiphospholipid syndrome (APS).

The diagnosis of Evans syndrome was established based on clinical evidence of autoimmune hemolytic anemia (AIHA) and immune thrombocytopenia (ITP), which developed after a recent parvovirus B19 infection. The patient was started on dexamethasone 40 mg for two days, but did not respond to steroid treatment. She was subsequently started on intravenous immunoglobulin (IVIG) treatment for three doses, and her platelet count gradually recovered before she was discharged home stable.

Parvovirus B19 may contribute to autoimmune disease onset and progression through mechanisms such as molecular mimicry, immune system disruption, and chronic infection. Some viral peptides of parvovirus B19 share similar epitopes with host antigens, and the cross-reactive antibodies produced by activated B lymphocytes also bind antigens on red blood cells and platelets, causing hemolysis and thrombocytopenia. Currently, consensus recommends steroids and IVIG as first-line and rituximab as the second-line treatment.

## Introduction

Evans syndrome is a rare autoimmune disorder characterized by the concurrent or sequential development of autoimmune hemolytic anemia (AIHA) and immune thrombocytopenia (ITP) [1]. The occurrence of Evans syndrome is considered a negative prognostic factor compared to isolated AIHA, especially as patients with Evans syndrome have a higher risk for thrombotic events due to hypercoagulability. Evans syndrome can be triggered by fungal infections, such as aspergillosis or tuberculosis, but is rarely caused by parvovirus B19 [2]. It was also reported as a paraneoplastic condition in patients with Hodgkin lymphoma or leukemia.

Parvovirus B19 is a single‐stranded DNA virus with tropism for bone marrow progenitor cells. It is known to cause aplastic crisis or pure red cell aplasia in susceptible individuals by binding P antigen (globoside) on erythroid progenitor cells, resulting in erythroid hypoplasia [3]. We present a case of Evans syndrome triggered by parvovirus B19 in a patient with pre-existing positive antiphospholipid antibodies.

## Case presentation

**Figure 1:**
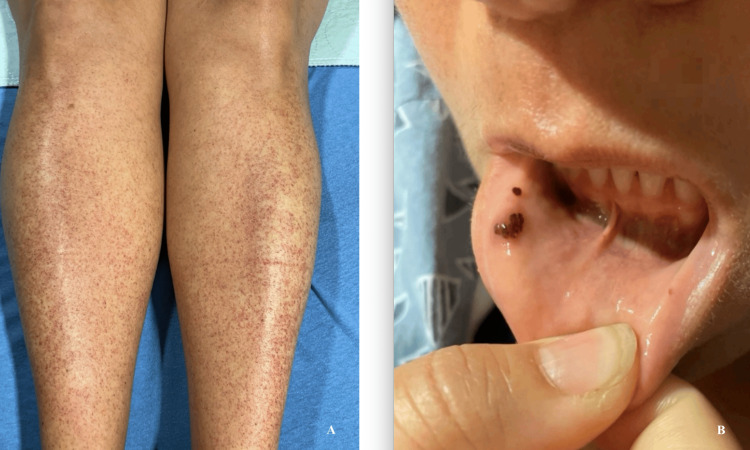
Images from the patient's physical examination reveal petechiae A: Pinpoint-sized petechiae on bilateral lower extremities; B: Mucosal petechiae in the oral cavity

A 47-year-old female with a past medical history of positive antiphospholipid antibodies presented with a petechial rash on the bilateral lower extremities for two days. Her symptoms started seven days after her child had slapped cheek disease. Physical examination revealed a rash on bilateral lower extremities and mucosal petechiae in the oral cavity (Figure 1).

The patient was otherwise hemodynamically stable. Initial labs showed anemia and critically low platelets of less than 2 x 103/uL. Other relevant lab findings included a low reticulocyte count percentage, elevated lactate dehydrogenase, and low haptoglobin (Table 1).

**Table 1:** Initial lab results on day one Labs revealed anemia with severe thrombocytopenia, positive hemolysis labs, and positive IgM and IgG of parvovirus B19. LDH: Lactate dehydrogenase

Parameter	Results	Normal range
WBC (10*3/uL)	6.6	4.40-10.50
Hemoglobin (g/dL)	9	11.4-14.7
Platelet ( 10*3/uL)	<2	139-361
Reticulocytes (%)	1.21	0.60-1.90
Haptoglobin (mg/dL)	<10	30-200
LDH (U/L)	366	60-200
IgM titer for parvovirus B19	25.58	<=0.89 IV
IgG titer for parvovirus B19	5.41	<=0.90 IV

The direct antiglobulin test (DAT) was positive for warm autoantibody, confirming the presence of autoimmune hemolysis. The peripheral smear revealed normocytic anemia with severe thrombocytopenia with scattered spherocytes. There was no evidence of lymphoma on flow cytometry. Coagulation studies, iron panel, vitamin B12 level, folate, ANA, complement level, ADAMTS13, HIV, and hepatitis were negative. Blood smear showed no schistocytes. The IgM titer of parvovirus B19 was very high, and the IgG titer was slightly elevated. The patient also tested positive for lupus anticoagulant and had positive antibodies for beta 2 glycoprotein and cardiolipin that were detected many years ago during an investigation for a false-positive syphilis test in her previous pregnancy. However, she never had thrombotic events or recurrent pregnancy loss that would fulfil the diagnosis of antiphospholipid syndrome (APS).

The differential diagnoses include systemic lupus erythematosus (SLE), thrombotic thrombocytopenic purpura, hemolytic uremic syndrome (HUS), APS, and hematologic malignancies. The patient did not have a rash, joint pain, pleural effusion, proteinuria, or any other clinical manifestations suggesting lupus and tested negative for antinuclear antibodies (ANA) and had a normal complement level, which made lupus highly unlikely. A negative ADAMTS 13, negative schistocytes, normal kidney function, and lack of thrombotic event ruled out HUS and thrombotic thrombocytopenic purpura (TTP). A normal lymphocyte count and lack of lymphadenopathy suggest a low concern of leukemia and lymphoma.

The diagnosis of Evans syndrome was established based on clinical evidence of AIHA and ITP, which developed after a recent parvovirus B19 infection. Bone marrow biopsy was not pursued due to the well-established clinical diagnosis and low clinical suspicion of hematological malignancy. The patient was started on dexamethasone 40 mg for two days, but did not respond to steroid treatment. She received one unit of platelet transfusion due to a persistent extremely low platelet count, which also failed. She was subsequently started on intravenous immunoglobulin (IVIG) treatment for three doses, and her platelet count gradually increased from 2 to 91 x 103/uL (139 - 361 10*3/uL). The patient recovered after IVIG treatment and was discharged home. She was followed up by a hematologist in the outpatient department and was started on a prednisone taper for persistent positive antiglobulin antibody. Her platelet count remained stable during outpatient follow-up.

## Discussion

To our knowledge, this is the fourth report of parvovirus B19-induced Evans syndrome in adults. Due to the rarity of this condition, it is important to raise the awareness of clinicians to recognize the association between Evans syndrome and parvovirus B19. Understanding the clinical implications of parvovirus B19 is crucial, as it can cause erythema infectiosum (fifth disease) and acute symmetric polyarthropathy in healthy populations and hydrops fetalis in pregnant women. In immunocompromised individuals, it is associated with pure red blood cell hypoplasia, pancytopenia/bone marrow suppression [2], vasculitis, and myocarditis [4]. Patients with pre-existing malignant thymoma may develop acute autoimmune hemolytic anemia and thrombocytopenia after parvovirus B19 infection, suggesting that patients with predisposing conditions such as dysregulated immune response due to malignancy or autoimmune condition [5] have a higher risk of developing Evans syndrome [6].

Even though parvovirus B19 is known to cause pure red blood cell hypoplasia, leukocytopenia and thrombocytopenia sometimes occur in addition to erythrocytopenia [7]. For example, a retrospective study showed 17 of 35 patients (49%) with ITP in the pediatric population had evidence of parvovirus B19 DNA in the peripheral blood, bone marrow, or both [8]. Rarely, parvovirus B19 is associated with hemophagocytic lymphohistiocytosis (HLH) [9,10] and thrombotic microangiopathy [11].

Currently, our understanding of the molecular pathway and pathogenesis of parvovirus-associated autoimmune reactions is limited. Patients presenting with concurrent thrombocytopenia and acute parvovirus B19 infection were found with an absence of erythroid progenitor cells in bone marrow aspiration [12] or had a marked reduction of bone marrow megakaryocytes [13]. There are three proposed theories on how parvovirus B19 contributes to autoimmune disease onset and progression through mechanisms such as molecular mimicry [14], immune system disruption [15], and chronic infection [16]. Some viral peptides, such as NS1, share similar epitopes with host antigens, and the cross-reactive antibodies produced by activated B lymphocytes triggered by parvovirus B19 also bind antigens on red blood cells and platelets, causing hemolysis and thrombocytopenia [17].

A study showed significantly higher positivity rates for anti-B19-VP1-unique region (VP1u) and anti-B19-nonstructural protein-1 (NS1) antibodies in patients with adult Still’s disease and lupus compared with healthy controls, and patients with positive sero-reactivity of anti-B19-VP1u and anti-B19-NS1 had a higher risk of developing cytopenia [18]. The theory of bystander activation is different from molecular mimicry and focuses on the disturbances of the internal environment after viral infection. The host cells undergo pathological apoptosis after being invaded by parvovirus B19 and release cellular content, including self-antigen, which amplifies pre-existing autoimmune processes in susceptible patients [19]. Another possible explanation is that cytokine production triggered by parvovirus B19 infection results in a chronic inflammatory status, which suppresses hematopoietic cell proliferation. In an observation study, peripheral blood mononuclear cells were retrieved from patients with recent parvovirus B19 infection and were stimulated by mitogen, which induced over-secretion of interferon-gamma (IFN-gamma), which is known to cause hematopoietic suppression; this could be a possible mechanism of parvovirus-induced reticulocytopenia and thrombocytopenia [20].

Our patient has had pre-existing positive lupus coagulants for many years but has not developed any clinical symptoms. The correlation of positive antiphospholipid antibodies and parvovirus B19 infection has been observed among patients with rheumatic diseases, and these two conditions also share overlap in clinical manifestations, including venous thromboembolism (VTE) and pregnancy loss. An increasing amount of evidence suggests a connection between parvovirus B19 and autoimmune diseases, and especially an association between B19-VP1u and APS [21]. Studies showed that the VP1 structural protein of parvovirus B19 contains a unique region with phospholipase A2-like activity that might trigger the production of anti-phospholipase antibodies [22]. In Tzang et al.'s study, 14 (58%) of 24 patients with positive anti-phospholipid antibodies showed IgG against VP1/VP2 and viral genomes, indicating the presence of acute or persistent infection. Autoantibodies against beta-2-glycoprotein and phospholipid in the serum of patients with parvovirus B19 infection were cross-reactive with viral structural protein B19-VP1u. Furthermore, mice immunized with anti-B19-VP1u antibody developed autoantibodies against beta-2 glycoprotein, phospholipids, the clinical syndrome of thrombocytopenia, and prolonged activated partial thromboplastin time (aPTT), which suggests that viral structural protein enhances cytokine production and might be directly involved in the onset or progression of autoimmune reactions mediated by antiphospholipid antibodies [23].

## Conclusions

It is important to learn from this case that parvovirus B19 infection should be considered in patients presenting with unexplained AIHA, Evans syndrome, or pancytopenia. Parvovirus B19 can co-exist with autoimmune diseases and can also be the trigger of autoimmune disease onset, possibly through the mechanism of molecular mimicry and bystander activation. The trajectory of parvovirus B19-triggered Evans syndrome varies from being self-limited in nature to lasting for months or being of the relapsing-remitting type. There has been no randomized trial to provide evidence on optimal treatment for Evans syndrome due to the rarity of this condition. In general, patients have a limited response to steroids but can improve after IVIG infusion.
